# N1, N12-Diacetylspermine Is Elevated in Colorectal Cancer and Promotes Proliferation through the miR-559/CBS Axis in Cancer Cell Lines

**DOI:** 10.1155/2021/6665704

**Published:** 2021-09-24

**Authors:** Teng Mu, Tingguang Chu, Wenxin Li, Qianze Dong, Yong Liu

**Affiliations:** ^1^School of Biomedical Engineering, Dalian University of Technology, Dalian, China; ^2^Liaoning Maidi Biotechnology, Benxi, Liaoning, China; ^3^Department of Pathology, College of Basic Medical Sciences and the First Affiliated Hospital, China Medical University, Shenyang, China; ^4^School of Life and Pharmaceutical Sciences, Dalian University of Technology, Panjin 124221, China

## Abstract

N1, N12-Diacetylspermine (DiAcSpm) has been reported to be upregulated in the urine of cancer patients. Mass spectrometry has shown elevated DiAcSpm expressions in colorectal cancer (CRC) tissues. However, the diagnostic application of DiAcSpm is not available due to a lack of diagnostic grade antibodies. Also, its biological roles in CRC cells remain unexplored. In the present study, we developed an antibody that directly detected DiAcSpm expression in paraffin-embedded tissues. We also characterized its biological characteristics and underlying mechanisms. Polyclonal antibodies were generated by immunizing animals with a synthetic product of DiAcSpm. Antibody DAS AB016 showed strong sensitivity against DiAcSpm in CRC tissues. Immunohistochemistry results showed that DiAcSpm expression was significantly elevated in CRC tissues. High levels of DiAcSpm correlated with the clinical stage and Ki67 index. DiAcSpm treatment increased levels of proliferation, cell cycle progression, and cyclin D1 and cyclin E proteins in CRC cell lines, SW480 and Caco-2. DiAcSpm also upregulated ATP production in these two cell lines. RNA-sequencing showed that DiAcSpm downregulated miR-559, which was confirmed using RT-qPCR. The luciferase reporter assay, western blotting, and RT-qPCR showed that *cystathionine β-synthase* (*CBS*) *was* the target of miR-559. miR-559 inhibited, while CBS accelerated, CRC proliferation. In addition, CBS siRNA knockdown blocked the biological effects of DiAcSpm on CRC cells. In conclusion, DiAcSpm was found to be increased in CRC tissues using a newly developed antibody. DiAcSpm accelerated CRC proliferation by regulating the miR-559/CBS axis.

## 1. Introduction

Colorectal cancer (CRC) is one of the most common malignancies and a leading cause of cancer-related death worldwide [1, 2]. Despite progress in treatment strategies in recent years, patient outcomes remain unsatisfactory, especially in patients with advanced-stage disease. Conventional therapies have failed to achieve long-term survival, emphasizing the need for new gene therapies for the treatment of CRC. Thus, it is essential to identify new biomarkers and to identify the molecular mechanisms of CRC progression.

N1, N12-Diacetylspermine (DiAcSpm) is a minor component of human polyamines which accounts for less than 0.5% of the total polyamines in normal urine [3]. DiAcSpm was found to be elevated in the urine of patients with early-stage cancers, including CRC, making it a potential tumor biomarker [4–6]. Using mass spectrometry, it has been reported that DiAcSpm is elevated in CRC tissue extracts using mass spectrometry [7]. DiAcSpm might be produced by cancer cells themselves or by infiltrating noncancer cells. It is thought that rapidly growing cells generally have increased intracellular polyamine levels and actively metabolize polyamines, and a previous study showed that peritoneal macrophages from tumor-bearing mice produced DiAcSpm [4]. To date, direct evidence for the production of DiAcSpm by cancer cells in vivo remains unknown due to a lack of in situ detection methods such as immunohistochemistry.

It has been reported that polyamine metabolites are essential for cell growth and development, and increased polyamine levels are associated with increased cell proliferation [8]. However, although polyamines are probably related to numerous cellular processes, the specific mechanisms underlying their modes of action have not been well defined. It has been shown that polyamines regulate specific gene expressions through both transcriptional and posttranscriptional processes [9]. Urinary DiAcSpm was significantly increased in nonsmall cell lung cancer, especially in squamous cell carcinoma. An increased urinary DiAcSpm value was significantly associated with pathological stage, other histological invasive factors and unfavorable outcomes in patients [3]. DiAcSpm levels were higher in CRC tissue and its liver metastasis than in adjacent normal tissues. The tumor/normal ratio was greater than 1.5 in 38% and 78% of low-grade intraepithelial neoplasia and high-grade intraepithelial neoplasia, respectively [7]. To date, most research has focused on its diagnostic and prognostic values, although its biological roles in cancer cells, including CRC, remain unexplored.

In the current study, we characterized DiAcSpm expression in human CRC tissues using a newly developed antibody. We also examined the biological roles of DiAcSpm in CRC cell lines. Our results suggested that DiAcSpm was upregulated in CRC and promoted cell proliferation.

## 2. Materials and Methods

### 2.1. Tissue Samples

The current study concerning clinical samples was approved by the institutional review board of China Medical University (Reference Number: 20170228), and the participants provided written informed consent. The present study was conducted by following the tenets of the Declaration of Helsinki [10]. The paraffin-embedded cancer tissue blocks were from the pathology archives at the First Affiliated Hospital of China Medical University, which contained specimens no longer required to be maintained.

### 2.2. Production of DAS Antibodies

A total of twelve adult female BALb/C mice (eight week old) were immunized intraperitoneally and subcutaneously with 100 *μ*g DAS antigen, which was completely emulsified using an equal amount of complete Freund's adjuvant (Sigma-Aldrich, St. Louis, MO, USA). The antigen development was based on a previous report [11]. The carrier protein BSA was reacted with S-acetyl-mercaptosuccinic acid to generate S-acetyl-mercaptosuccinic acid (AMS)-BSA complex. The bivalent cross-linking agent N-(4-maleimidobutyryloxy) succinimide (GMBS) is added to form the AcSpm-GMB-BSA antigen. The mice were boosted twice at 3-week intervals and in three consecutive days before cell fusion. Three days after the last boost of DAS antigen (100 *μ*g), we fused the splenocytes of the immunized BALb/C mice with SP2/0 myeloma cells using 50% polyethylene glycol 1500 (Roche, Indianapolis, IN, USA). We then selected hybridomas in RPMI-1640 medium containing thymidine (Sigma-Aldrich) [12]. An ELISA was used to identify positive clones, and we established and characterized hybridoma-producing mAbs after three subclone selections.

### 2.3. Immunohistochemistry

The process of FFPE tissue preparation includes 5 steps: fixation, dehydration, clearing, paraffin infiltration, and embedding. Briefly, the tissue was immersed for approximately twenty-four hours in a 10% neutral buffered formalin. The tissue was then dehydrated using increasing concentrates of ethanol (70% 15 min, 90% 15 min, 100% 15 min, 100% 30 min, and 100% 45 min). Next, the tissue is immersed in xylene (clearing). Then the tissue was placed into liquid paraffin. After being thoroughly infiltrated with paraffin, the specimen is embedded into a block.

4 *μ*m paraffin slides were produced and treated with xylene and alcohol. H_2_O_2_ was applied to slides to block peroxidase activity. The slides were incubated with antibodies against DiAcSpm (1 : 300) and Ki67 (1 : 400; Abcam, Cambridge, MA, USA) overnight at 4°C. An Elivision Plus Kit and DAB + Kit (MaiXin, Fuzhou, China) were used for immunohistochemical staining. Hematoxylin was used for counterstaining. Negative control experiments were performed by incubating IgG antibodies on slides.

Immunostaining was evaluated by two pathologists from the pathology department in the First Affiliated Hospital of China Medical University (Qianze Dong and Lin Fu). Immunostaining with DiAcSpm was evaluated by combining staining intensity and percentage. DiAcSpm staining intensity was classified with three degrees including 0 (negative), 1 (moderate), and 2 (strong). The percentage was scored as 1: 1%–25%, 2: 26%–50%, 3: 51%–75%, and 4: 76%–100%. These two scores were multiplied to get the final score. The slides were determined at a DiAcSpm low level with a final score <4. Slides with scores ≥4 were determined as a high level of DiAcSpm.

For analysis of the Ki67 index, tumors exhibiting a Ki67 index ≥50% were considered as highly proliferative/Ki67 high expression. Based on the cutoff value, 60 of the 96 CRC specimens were considered to have highly proliferative potential.

### 2.4. Cell Culture and Treatment

SW480 and Caco-2 cell lines were purchased from the American Type Culture Collection (Manassas, VA, USA). The cells were maintained in a DMEM culture medium with 10% fetal bovine serum. The plasmid was transfected by Lipofectamine 3000 reagent (Invitrogen, Carlsbad, CA, USA). CBS-specific siRNA was transfected using Dharmafect1 reagent (Dharmacon, Lafayette, CO, USA).

### 2.5. Cell Counting Kit-8 (CCK-8) Assay

The relative cell variability was examined using CCK-8 (Dojindo, Kumamoto, Japan). Cultured cells were plated into flat 96-well plates. Ten microliters of CCK8 reagent were then added to 100 *μ*L of culture medium in the plates, followed by incubation for 2-3 h at 37°C. The plates were removed from the incubator and the absorbance was measured using a microplate reader (Bio-Rad, Hercules, CA, USA) at 450 nm.

### 2.6. Real-Time Quantitative PCR (RT-qPCR) and RNA Sequencing

RNA from cells was obtained using TRIzol reagent and quantified by determining the A260/A280 ratio using a nanodrop spectrometer. The cDNA reverse transcription was conducted using RT MasterMix (Takara, Shia, Japan). Real-time PCR amplification was conducted using the SYBR-Green Master-Mix Kit (Takara) on an ABI-7500 real-time PCR system (Applied Biosystems, Foster City, CA, USA). The miRNA was normalized to U6. CBS was normalized to glyceraldehyde 3-phosphate dehydrogenase (GAPDH). Calculation of the relative gene expression level was performed using the 2^−△△ct^ method [13]. Experiments were conducted in triplicate.

The RNA-seq experiments were performed by Novogene Corporation (Beijing, China). The libraries were sequenced on an Illumina NovaSeq6000 platform. The sequence data were subjected to standard quality control (QC). The clean reads percentage for 2 samples was 98.85% (SW480 control) and 98.96% (SW480 DiAcSpm), which were used for further analysis.

### 2.7. Enzyme-Linked Immunosorbent Assay (ELISA)

ELISA was performed using a kit based on the DiAcSpm antibody (Liaoning Maidi Biotechnology, Benxi, Liaoning, China) (Patent Number: CN105527434A). 100 *μ*l pure antigen (concentration 2 mg/ml) was added in each well of the ELISA compatible 96-well plate which was incubated overnight at 4°C. Wash the plate three times in wash buffer. Add 150 *µ*l of blocking solution to each well. Incubate for 2 hours at 37°C. Wash three times in wash buffer. Prepare the antigen antibody mixture by adding 50 *µ*l of antigen to 50 *µ*l of antibody for each well in the assay. Incubate for 1 hour, and then add 100 µl of the mixture to each well. Incubate for 1 hour at 37°C. After washing, add 100 *µ*l enzyme-conjugated secondary antibody to each well. Incubate for 1 hour. Wash 3 times in wash buffer. Add 100 *µ*l of the substrate solution to each well and incubate at room temperature for 15 minutes. Then 50 *µ*l stop buffer was added. Then, the plate was detected using a plate reader (Bio-Rad).

### 2.8. Western Blotting

Cells were lysed using a lysis buffer from Thermo Fisher Scientific (Waltham, MA, USA). A total of 50 *μ*g of protein was used for SDS-PAGE. Protein was transferred onto polyvinylidene difluoride membranes, which were incubated with 5% milk at room temperature for 2 h to reduce the nonspecific background. The membranes were incubated with antibodies against cyclin D1, cyclin E, CBS (1 : 1,200; Cell Signaling Technology, Danvers, MA, USA), and GAPDH (1 : 3,000; Cell Signaling Technology) for at least 10 h. Then, the membranes were washed with TBS-T and incubated with 1 : 2,000 secondary antibodies (Thermo Fisher Scientific) for 2 h. After washing, the fluorescence on the membranes was developed using the ECL Reagent Kit (Thermo Fisher Scientific), and the images were documented using a DNR Bio-Imaging System (DNR Bio-Imaging, N.eve Yamin, Israel).

### 2.9. Cell Cycle Analysis

A 0.5% trypsin solution was used to dissociate cells from culture plates. Binding buffer (250 *μ*L) was used for the resuspension of cells. For the cell cycle assay, the cells were fixed in 1% paraformaldehyde and stained with 5 mg/mL propidium iodide, and analyzed by flow cytometry.

### 2.10. ATP Level Measurement

For ATP measurements, the cells were lysed in 200 *μ*L of ATP Assay Buffer (Beyotime, Guangzhou, China), and the lysates were centrifuged at 12,000 rpm for 5 min. A total of 100 *μ*L of ATP reaction solution was added to a 96-well plate and 20 *μ*L of the sample was added to the ATP reaction solution. A standard curve was developed and the fluorescence was measured using a microplate reader.

### 2.11. Luciferase Reporter Assays

Wild-type and mutant sequences of CBS 3′-UTR with the putative miR-559 binding site were synthesized and subcloned into the luciferase vector to generate wild-type and mutant luciferase reporters [14]. The wild-type miR-559 target site in CBS 3′- UTR was UUACUUU, which started at the 1,664 bp of the CBS 3′UTR region. The mutant miR-559 target site was UUCCCUU. Then, 100 ng of the luciferase vector was cotransfected with the miR-559 mimic or negative control using Lipofectamine 3000. Luciferase intensity was examined using the dual-luciferase Reporter Assay Kit (Promega, Madison, WI, USA).

### 2.12. Statistical Analysis

SPSS 18.0 software (SPSS, Chicago, IL, USA) was used for statistical analyses. Correlations between DiAcSpm expressions and clinical parameters were analyzed using *χ*2 tests. The significance between different groups was examined using Student's *t*-test. A value of *P* < 0.05 was assumed to be statistically significant.

## 3. Results

### 3.1. Expression of DiAcSpm in CRC Specimens

**Figure 1:**
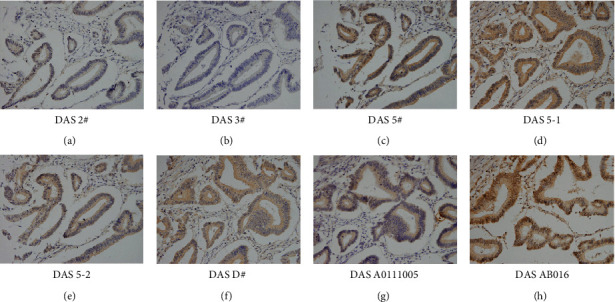
Screening of DiAcSpm antibodies. (a–h) DiAcSpm staining of CRC tissue using eight different antibodies. Note that DAS AB016 showed the highest sensitivity.

**Figure 2:**
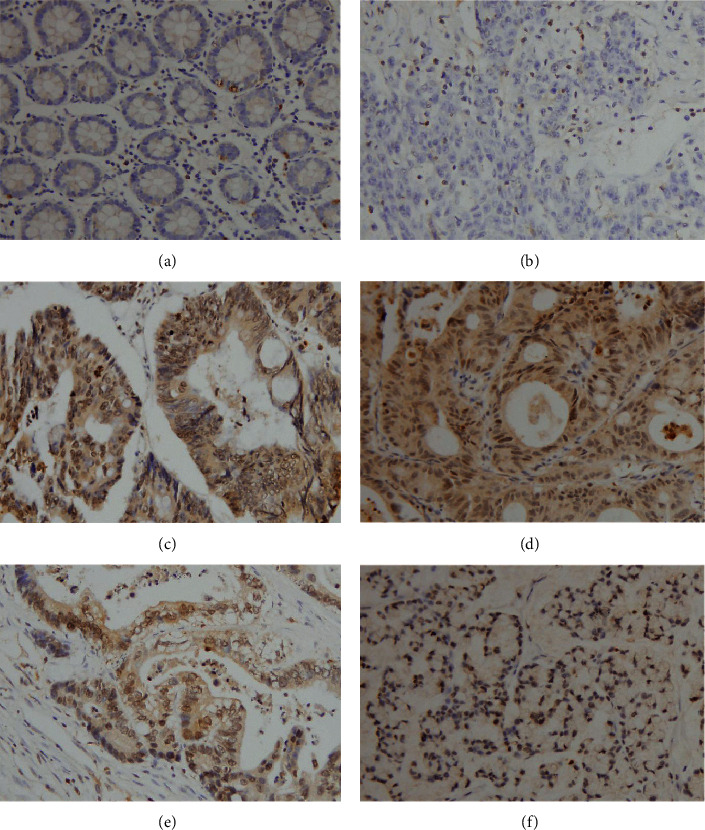
Expression of DiAcSpm in CRC. (a) Negative staining of DiAcSpm in normal colon tissue. (b) Negative DiAcSpm staining of CRC tissues. (c) Positive DiAcSpm staining in an adenocarcinoma. (d) Positive cytoplasmic and nuclear DiAcSpm staining in a papillary adenocarcinoma. (e) Moderate cytoplasmic and nuclear DiAcSpm staining in a mucinous adenocarcinoma. (f) Strong nuclear DiAcSpm staining in a signet ring cell carcinoma.

We developed and tested eight antibodies their sensitivities in CRC tissues. Immunohistochemistry was performed in the same CRC tissue sections using these antibodies at a dilution of 1 : 300. As shown in Figures 1(a)–1(h), antibody DAS AB016 showed the best sensitivity. We compared the immunosensitivity and staining signal localization of DAS 5-1 and DAS AB016. As shown in Supplementary Figure 1A, staining signals of both antibodies showed cytoplasmic and nuclear localization. DAS DAS AB016 showed higher sensitivity than DAS 5-1 (Supplementary Figure 1B). To eliminate the possibility that the nuclear staining by DAS AB016 antibody may be an artifact. We used negative controls by incubating tissue slides with DAS AB016 together with excess DiAcSpm, which was used to mask the reactivity toward DiAcSpm in the specimen without interfering the IgG portion of the anti-DAS antibody (Supplementary Figure 2). Control slides showed negative immunostaining compared with slides incubated with DAS AB016 alone. We have also tested its specificity in several polyamines using ELISA. Using the standard curve of the DiAcSpm antibody as a standard, we examined the concentration of 4 reference materials including spermine (10 *μ*m), putrescine(10 *μ*m), diacetylspermidine (5 *μ*m), and L-tyrosine (2 mM) for three times. The results are listed in Supplementary Tables 1–3. These results indicated that our antibody has high specificity and has low cross-reactivity with related polyamine species. We then tested DiAcSpm expressions in 96 CRC tissues and 16 normal colon tissues using DASAB016.  Figure 2 indicates that DiAcSpm showed negative/weak staining in normal colon tissues. DiAcSpm expression was elevated in tumor tissues. In 96 cases, 67% (65/96) showed high DiAcSpm expressions.  Table 1 shows that high DiAcSpm levels significantly correlated with an advanced tumor node metastasis state (*P*=0.0022), positive nodal metastasis (*P*=0.038), and high Ki67 proliferation index (*P*=0.0041). These results showed that DiAcSpm was elevated in human CRC and was associated with their malignant features.

### 3.2. DiAcSpm Promotes Proliferation in CRC Cell Lines

**Figure 3:**
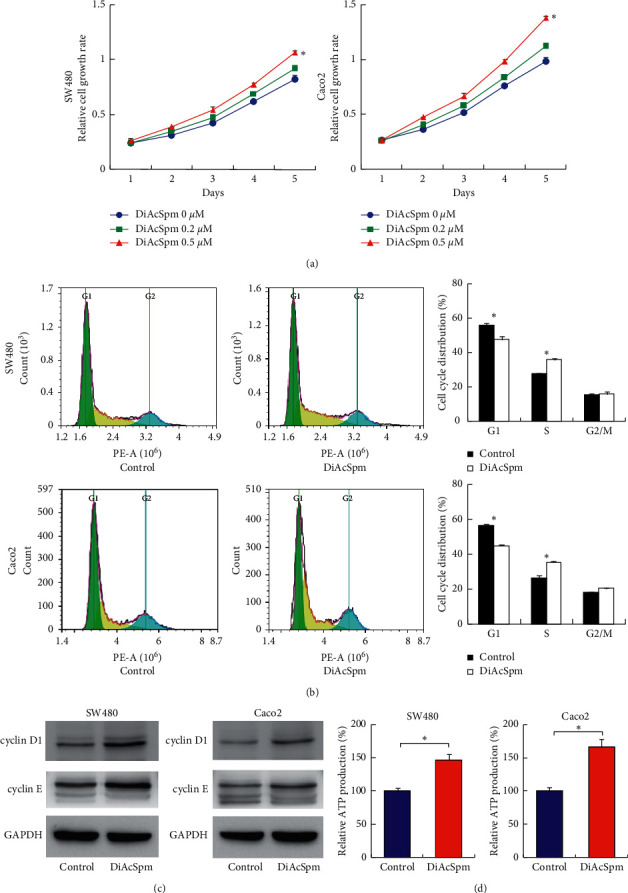
DiAcSpm promotes proliferation. (a) The CCK-8 assay showed that DiAcSpm increased the growth rate at a concentration of 0.2 and 0.5 *μ*m in SW480 and Caco-2 cell lines (^*∗*^*P* < 0.05 DiAcSpm 0.5 *μ*m vs. control). (b) Flow cytometry showed that DiAcSpm treatment (0.5 *μ*m) increased the S phase population and decreased the G1 phase population in both SW480 and Caco-2 cell lines (^*∗*^*P* < 0.05 DiAcSpm vs. control). (c) Western blotting showed that DiAcSpm treatment upregulated protein expressions of cyclin D1 and cyclin E. (d) The ATP assay showed that DiAcSpm treatment increased the ATP production rate in both SW480 and Caco-2 cell lines (^*∗*^*P* < 0.05 DiAcSpm vs. control).

To examine the biological roles of DiAcSpm in CRC cells, we treated SW480 and Caco-2 cell lines with different concentrations of DiAcSpm for five days. The CCK-8 assay showed that DiAcSpm significantly increased the growth rate at a concentration of 0.5 *μ*m (Figure 3(a)). We used ELISA to determine the intracellular levels of DiAcSpm in CRC cells before and after treatment. ELISA was carried out in SW480 and Caco-2 cell lysates 24 hours after 0.5 *μ*m DiAcSpm treatment. As shown in Supplementary Figure 3, DiAcSpm treatment significantly increased its intracellular levels in both cell lines. We conducted cell cycle analysis using flow cytometry.  Figure 3(b) shows that DiAcSpm treatment increased the S phase population with a concomitant decrease in the G1 phase population in both SW480 and Caco-2 cells. Accordingly, DiAcSpm upregulated protein expression of the cell cycle regulators, cyclin D1 and cyclin E (Figure 3(c)). ATP is essential for cancer cell survival and malignant growth. We, therefore, determined if DiAcSpm regulated energy production in CRC cells.  Figure 3(d) shows that DiAcSpm treatment increased the energy (ATP) production in both SW480 and Caco-2 cell lines. Together, these data suggested that DiAcSpm promoted CRC proliferation, possibly by facilitating cell cycle transition and energy metabolism.

### 3.3. DiAcSpm Downregulates miR-559 and Upregulates CBS in CRC Cells

**Figure 4:**
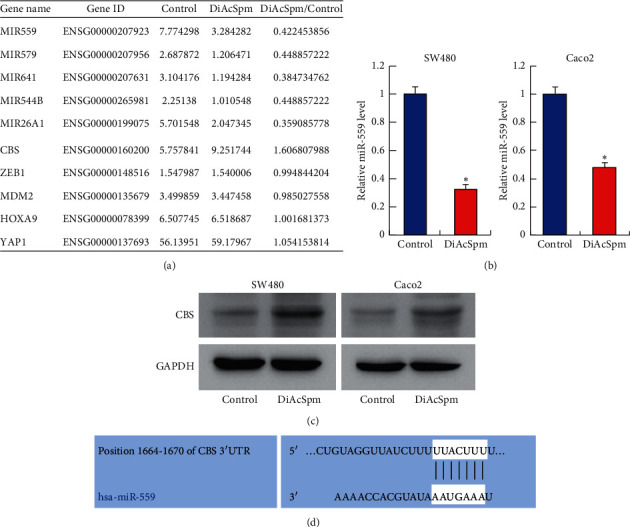
DiAcSpm downregulates miR-559 and upregulates cystathionine *β*-synthase (CBS). (a) RNA-sequencing results showed that miR-559 was one of the top downregulated miRNAs after DiAcSpm treatment in SW480 cells. DiAcSpm treatment also upregulated CBS mRNA expression. (b) Real-time PCR showed that DiAcSpm downregulated miR-559 in both SW480 and Caco-2 cell lines (^*∗*^*P* < 0.05 DiAcSpm vs. control). (c) Western blotting showed that DiAcSpm treatment upregulated CBS protein. (d) TargetScan revealed that there was a potential binding site of miR-559 on the CBS 3′-UTR region.

To identify its underlying mechanisms, we treated SW480 cells with 0.5 *μ*m DiAcSpm for two days and used RNA-sequencing (RNA-seq) to profile the global mRNA/miRNA change induced by DiAcSpm. RNA-seq showed that DiAcSpm induced a significant change of miRNAs in SW480 cells. The miR-559 was one of the top five most significantly changed miRNAs (Figure 4(a)). RT-qPCR was used to confirm the downregulation of miR-559 in both cell lines (Figure 4(b)). RNA-seq also showed that CBS, a potential tumor growth promoter, was one of the top upregulated genes (Figure 4(a)). There was no significant change of potential miR-641 targets ZEB1, HOXA9, MDM2, and YAP1(Figure 4(a)). In addition, top upregulated (>2-folds) and downregulated (<0.5-folds) genes and miRNAs are listed in Supplementary Tables 4 and 5. Western blotting also confirmed the upregulation of CBS after DiAcSpm treatment (Figure 4(c)). TargetScan prediction showed that there was a potential binding site of miR-559 on the 3′-UTR region of CBS (Figure 4(d)). Together, these results suggested that DiAcSpm might upregulate CBS by inhibiting miR-559.

### 3.4. miR-559 Inhibits Proliferation and Targets CBS in CRC Cells

**Figure 5:**
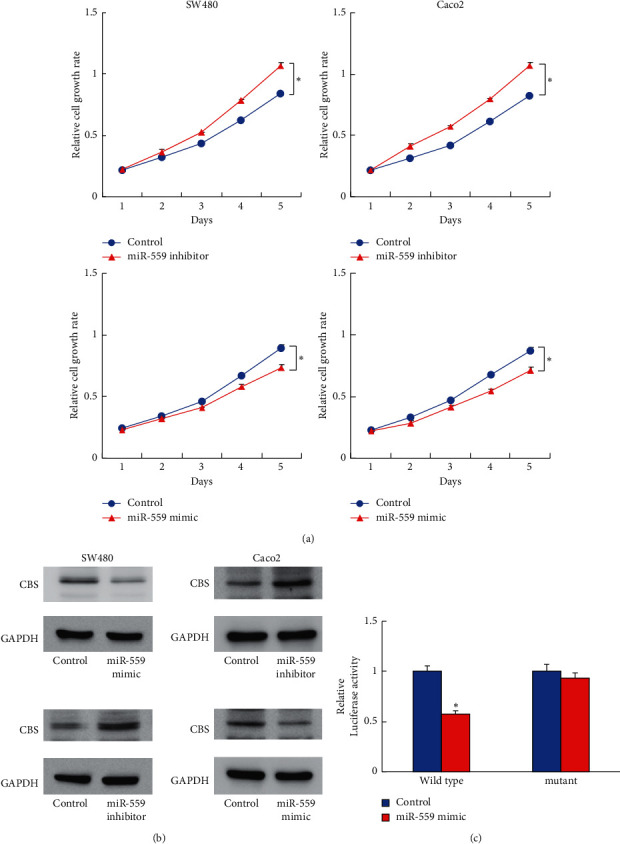
miR-559 inhibits proliferation and targets CBS in CRC cells. (a) The CCK-8 assay showed that a miR-559 mimics downregulated cell proliferation, while a miR-559 inhibitor upregulated cell proliferation in SW480 and Caco-2 cells (^*∗*^*P* < 0.05 miR-559 mimic/inhibitor vs. control). (b) Western blotting showed that miR-559 mimics downregulated CBS protein expression. A miR-559 inhibitor upregulated CBS protein expression in both SW480 and Caco-2 cell lines. (c) The luciferase reporter assay showed that cotransfection of a miR-559 mimic and wild-type CBS downregulated luciferase activity in SW480 cells (^*∗*^*P* < 0.05 miR-559 mimic vs. control).

To characterize the biological roles of miR-559 in CRC cells, we transfected a miR-559 inhibitor and mimicked it into both cell lines and measured the changes of cell proliferation.  Figure 5(a) shows that the miR-559 mimics downregulated cell proliferation in SW480 and Caco-2 cells, while the miR-559 inhibitor upregulated cell proliferation in both cell lines. To confirm the potential regulatory role of miR-559 on CBS, we measured the protein expression of CBS in cells transfected with the miR-559 mimic and inhibitor.  Figure 5(b) shows that the miR-559 mimic downregulated CBS protein expression, and the miR-559 inhibitor upregulated CBS protein expression. The luciferase reporter assay was then conducted to confirm their relationship. The miR-559 mimic and wild-type CBS or mutant reporter plasmid were cotransfected into SW480 cells. The miR-559 mimic downregulated luciferase activity in cells cotransfected with wild-type CBS reporter, but there was no significant change of activity in cells cotransfected with the mutant CBS reporter (Figure 5(c)).

### 3.5. CBS Promotes Proliferation in CRC Cells

**Figure 6:**
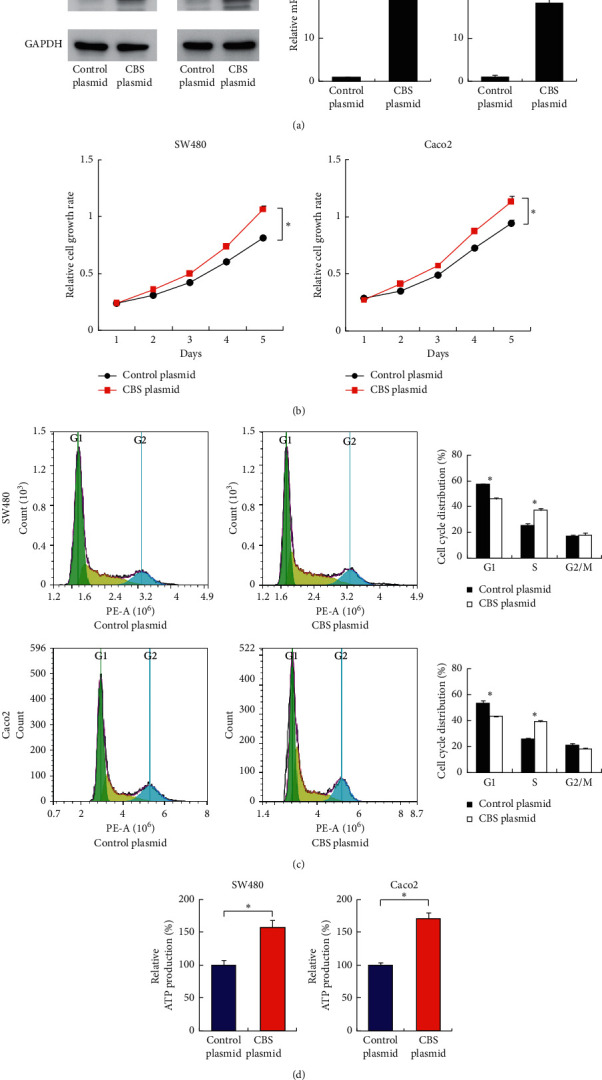
CBS promotes proliferation in CRC cells. (a) Western blots showed that transfection of a CBS plasmid upregulated its protein levels in both SW480 and Caco-2 cell lines. RT-qPCR showed that CBS transfection upregulated its endogenous mRNA levels. (b) The CCK-8 assay showed that CBS overexpression significantly increased growth in both SW480 and Caco-2 cell lines (^*∗*^*P* < 0.05 CBS plasmid vs. control plasmid). (c) Cell cycle analyses showed that CBS overexpression increased the S phase population and decreased the G1 phase population in both SW480 and Caco-2 cell lines (^*∗*^*P* < 0.05 CBS plasmid vs. control plasmid). (d) The ATP production assay showed that CBS overexpression increased the ATP production in both SW480 and Caco-2 cell lines (^*∗*^*P* < 0.05 CBS plasmid vs. control plasmid).

To confirm the biological role of CBS in CRC cells, we transfected the CBS plasmid into SW480 and Caco-2 CRC cells. The transfection efficiency was confirmed using western blotting and RT-qPCR (Figure 6(a)). The CCK-8 assay showed that CBS significantly increased the growth rate in both cell lines (Figure 6(b)), and cell cycle analysis showed that CBS overexpression facilitated cell cycle progression (Figure 6(c)). CBS overexpression also increased ATP production in both SW480 and Caco-2 cell lines (Figure 6(d)). Together, these results suggested that CBS promoted CRC proliferation and served as a downstream target of DiAcSpm.

### 3.6. DiAcSpm Regulates Proliferation by Targeting Upregulated CBS

**Figure 7:**
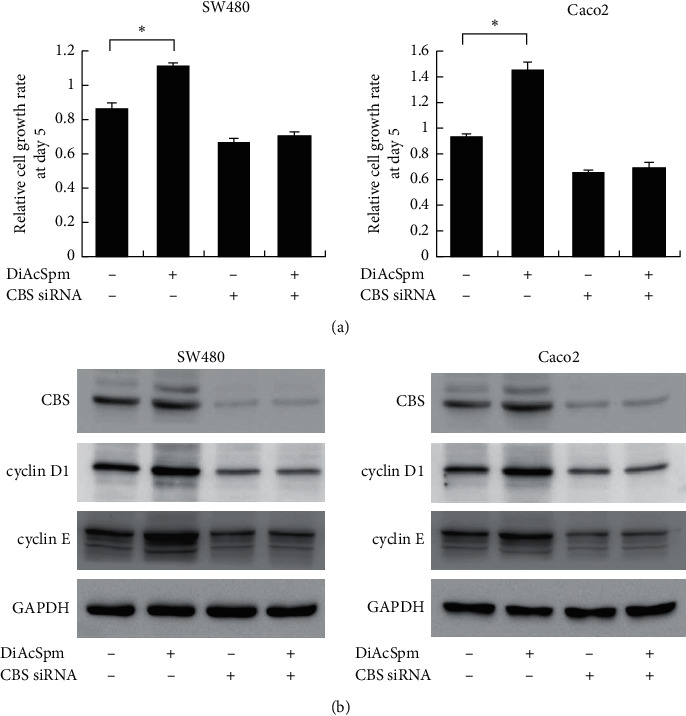
DiAcSpm regulates proliferation by targeted upregulating cystathionine *β* synthase (CBS). (a) The CCK-8 assay showed that the growth-promoting effect of DiAcSpm was abolished in SW480 and Caco-2 cells treated with CBS siRNA (^*∗*^*P* < 0.05 DiAcSpm+/CBS siRNA− vs. DiAcSpm−/CBS siRNA−). (b) Western blotting showed that the effects of DiAcSpm on cyclin D1 and cyclin E upregulation were partially abolished in SW480 and Caco-2 cells treated with CBS siRNA.

**Figure 8:**
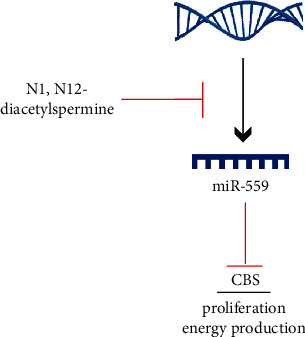
Schematic sketch explaining the possible mechanism of DiAcSpm in CRC cells. A schematic drawing of DiAcSpm downregulated miR-559, which targets and downregulates CBS in CRC cells.

To further confirm whether DiAcSpm regulated biological functions by upregulating CBS, we used CBS siRNA and DiAcSpm together in CRC cells and tested its effects on cell proliferation. Using the CCK-8 assay, Figure 7(a) shows that CBS siRNA inhibited cell proliferation. The growth-promoting effect of DiAcSpm was significantly diminished in cells treated with CBS siRNA. These results indicated that the growth-promoting effect of DiAcSpm was mediated, at least partially, by CBS in CRC cells. We also examined changes in cell cycle proteins (Figure 7(b)). Western blotting showed that the effects of DiAcSpm on cyclin D1 and cyclin E upregulation were abolished by CBS knockdown. A schematic drawing of the possible mechanism is shown in Figure 8.

## 4. Discussion

For the first time, we generate antibodies by immunizing mice with a synthetic product of DiAcSpm, which could directly detect DiAcSpm expression in paraffin-embedded tissues. Using our newly developed antibody, we showed that DiAcSpm was upregulated in human CRC cells in situ. DiAcSpm expression was elevated in 67% of CRC tissues examined, suggesting DiAcSpm as a biomarker for CRC screening and early diagnosis. In addition, DiAcSpm upregulation correlated with the Ki67 proliferation index and advanced clinical stage.

A previous study has shown a competitive immunochromatographic method for DiAcSpm determination by using selective antibody [15], and elevation of DiAcSpm has also been shown in various cancers, including CRC. However, in situ detection of DiAcSpm by immunohistochemistry in cancer tissues has not been reported due to a lack of specific antibodies. This is the first report showing DiAcSpm upregulation using antibody-based immunohistochemistry in paraffin-embedded tissues. Many cells in cancer patients produce DiAcSpm, including infiltrating noncancerous cells such as macrophages and fibroblasts. These noncancerous cells might also contribute to the increase of DiAcSpm in tumor tissues and urine of cancer patients. Our findings excluded the possibility that DiAcSpm in cancer tissues was mainly produced by infiltrating noncancer cells and showed that CRC cells were primarily responsible for DiAcSpm production. DiAcSpm could be used as a tumor biomarker in diagnosis because none of the normal tissues showed positive immunostaining. Our results also showed that DiAcSpm levels correlated with stage and Ki67 proliferation index, suggesting the potential use of DiAcSpm as a clinical indicator of malignant proliferation.

Polyamines have been reported to be involved in tissue proliferation and development, especially in the gastrointestinal tract. Previous studies have shown that polyamine-synthesis inhibitors disrupted normal intestinal development [16]. Polyamine metabolism also contributes to the mucosal homeostasis in the intestine and colon [17]. Polyamines have been associated with cancer, but their effect on numerous processes in carcinogenesis is poorly understood. Increased polyamine levels are associated with increased cell proliferation and decreased apoptosis with a change of related gene expression. Conversely, suppression of polyamine levels is associated with decreased cell growth and increased apoptosis [18, 19]. Although polyamines are necessary for angiogenesis during tumor growth [20], the underlying mechanisms are not well defined.

To date, the biological roles of DiAcSpm in human CRC remain elusive. We tested its effect on CRC cell proliferation using the CCK-8 assay and showed that DiAcSpm was able to promote CRC cell growth under certain concentrations. DiAcSpm treatment increased the S phase population percentage with a concomitant decrease in the G1 phase population in both SW480 and Caco-2 cells, which indicates that DiAcSpm treatment facilitating the transition from G1 phase to S phase of CRC cells. This was also supported by the result that DiAcSpm could upregulate cyclin D1 and cyclin E proteins. Because polyamines have been shown to be associated with cancer metabolism [21], we tested ATP production after DiAcSpm treatment and found that DiAcSpm upregulated ATP production in CRC cells. Overall, these results indicated that DiAcSpm was a positive regulator of CRC cell growth and metabolism.

Polyamines have been implicated in cellular functions such as nucleic acid and chromatin structure maintenance and protein synthesis [22, 23]. Experimental and modeling data demonstrate that natural polyamines can affect DNA conformation and aggregation [24]. Thus, DiAcSpm may play a potential role in cancer-related gene regulation.

The miRNAs are a class of small, endogenous, noncoding, single-stranded RNAs that bind to the 3′-UTR complementary sequences of their target mRNAs, resulting in mRNA degradation or inhibition of its translation [25]. Recently, miRNA dysregulation has been implicated in carcinogenesis, including CRC. The functions of these dysregulated miRNAs appear to be those of an oncogene and tumor suppressor, depending on the cellular environment. There are many tumor-suppressive miRNAs in CRC. For example, let-7a, which is significantly associated with better survival outcomes in CRC patients [26], has been shown to play a role in CRC progression by targeting oncogenes such as RAS and c-Myc.

Using RNA-seq, we determined the global changes of mRNAs and miRNAs and found the downregulation of miR-559, a potential tumor-suppressive miRNA, and upregulation of CBS, a cancer-related gene, after DiAcSpm treatment. The regulation of miR-559 on CBS in CRC cells was further validated using a reporter assay. The miR-559 has been reported as a tumor-suppressive miRNA in several cancers. It inhibits proliferation and invasion of gliomas [27, 28] and inhibits cancer aggressiveness and epithelial-mesenchymal transition in gastric cancers and papillary thyroid carcinomas [29, 30]. It also suppresses the proliferation and invasion of hepatocellular carcinomas in vitro [31]. We used RNA-seq to screen potential miRNAs and mRNAs regulated by DiAcSpm. Our RNA-seq results suggested that miR-559 was one of the top downregulated miRNAs after DiAcSpm treatment, where it functioned as a tumor-suppressive miRNA by targeting CBS in CRC cells.

CBS is the first and rate-limiting enzyme of the reverse transsulfuration pathway, the primary metabolic pathway for the synthesis of cysteine, which is used for the biosynthesis of proteins, glutathione, and the gaseous transmitter, hydrogen sulfide [32]. Recently, CBS has been reported to be overexpressed in CRC tissues and cell lines [33]. CBS promotes colon carcinogenesis by inducing cells to switch to an anabolic metabolism with increased production of energy [34]. CBS also regulates bioenergetics and mitochondria of ovarian cancer cells by increasing oxygen consumption and ATP generation [12]. Silencing and inhibition of CBS reduced CRC cell proliferation, ATP turnover, and glycolysis [33]. These results indicated that CBS functioned as an oncoprotein in CRC by regulating energy metabolism, which was consistent with our results, suggesting the role of DiAcSpm in ATP production and cancer growth might depend on its regulation of the miR-559/CBS axis. To confirm if the effect of DiAsSpm depended on CBS, CBS siRNA, and DiAcSpm were used to treat cells simultaneously. We found that CBS depletion partially abolished the positive effects of DiAcSpm on cell growth and cell cycle regulator proteins. These results suggested that the biological role of DiAcSpm was dependent, at least partially, on its regulation of CBS in CRC.

We also noticed that miR-641 was among the top downregulated miRNAs. miR-641 has been reported to be a tumor-suppressive miRNA by targeting various downstream genes, including ZEB1 [35], HOXA9 [36, 37], MDM2 [38], and YAP1 [39]. There was no significant change of these genes in SW480 cells based on our RNA-seq data. In addition, among the top upregulated miRNAs, miR-106b has been reported to be correlated with malignant behavior of CRC cells by targeting FJX1 [40] and SLAIN2 [41], the levels of which were not changed after DiAsSpm treatment, suggesting miR-641 and miR-106b might not be the major contributors to the proliferative property of CRC cells after DiAcSpm treatment. Moreover, analysis of top upregulated (>2-folds) and downregulated (<0.5-folds) genes did not show candidate genes that have been reported to be cancer related. However, we could not exclude the possibility that DiAcSpm regulates CRC proliferation through other mechanisms, which needs further investigation.

In conclusion, this study showed DiAcSpm overexpression in CRC cells using a newly developed antibody. Our study also revealed a novel role of DiAcSpm by linking its regulation of miR-559/CBS with CRC cell growth. This study provided novel insights into the mechanisms of miR-559/CBS in DiAcSpm-induced CRC progression and suggested their potential therapeutic uses. Further investigation is warranted to explore the upstream pathways of DiAcSpm production and the possibility of reducing DiAcSpm production.

## Figures and Tables

**Table 1:** Distribution of DiAcSpm status in CRC according to clinicopathological characteristics.

Characteristics	Number of patients	DiAcSpm low expression	DiAcSpm high expression	*P*
Age				
<60	39	10	29	0.2490
≥60	57	21	36	
Gender				
Female	41	13	28	0.9158
Male	55	18	37	
TNM stage				
I + II	56	25	31	0.0022
III + IV	40	6	34	
Tumor status				
T1 T2	26	9	17	0.7667
T3 T4	70	22	48	
Nodal status				
Negative	67	26	41	0.0380
Positive	29	5	24	
Differentiation				
Poor	16	2	14	0.065
Moderate	52	22	30	
Well	9	2	7	
Ki-67				
Low expression	36	18	18	0.0041
High expression	60	13	47	

## Data Availability

The data that support the findings of this study are available from the corresponding author upon request.

## Supplementary Materials

Supplementary Materials Supplementary Figure 1: comparison of antibody DAS 5-1 and DAS AB016. A. Higher resolution image showing immunoreactivity of both DAS 5-1 and DAS AB016 in the nucleus of CRC cells. B. Comparison of IHC staining activity of DAS 5-1 and DAS AB016 in one normal colon tissue and four CRC tissues. These 2 antibodies showed a similar staining pattern, although DAS AB016 showed higher sensitivity. Supplementary Figure 2: negative control using DiAcSpm to mask the reactivity of DAS AB016 toward DiAcSpm. DiAcSpm staining in 8 CRC specimens using DAS AB016 and negative control antibody using the same concentration of DAS AB016 combined with excess DiAcSpm. Supplementary Figure 3: intracellular level of DiAcSpm after treatment. SW480 and Caco-2 cell lines were treated with 0.5 *μ*m DiAcSpm for 24 hours. 10 cm dishes of treated and untreated (control) cells were collected using 100 *μ*L lysis buffer. Concentrations of DiAcSpm in different cell lysates were determined using ELISA. Supplementary Table 1: analysis of DiAcSpm antibody specific using ELISA (first). Supplementary Table 2: analysis of DiAcSpm antibody specific using ELISA (second). Supplementary Table 3: analysis of DiAcSpm antibody specific using ELISA (third). Supplementary Table 4: top upregulated genes and miRNAs after DiAcSpm treatment. Supplementary Table 5: top downregulated genes and miRNAs after DiAcSpm treatment.
