# Supplementary material for: N1, N12-Diacetylspermine Is Elevated in Colorectal Cancer and Promotes Proliferation through the miR-559/CBS Axis in Cancer Cell Lines
Source: J Oncol. 2021 Sep 24;2021:6665704. doi: 10.1155/2021/6665704 (PMC8486517; doi:10.1155/2021/6665704)
Supplement: Supplementary Materials — Supplementary Figure 1: comparison of antibody DAS 5-1 and DAS AB016. A. Higher resolution image showing immunoreactivity of both DAS 5-1 and DAS AB016 in the nucleus of CRC cells. B. Comparison of IHC staining activity of DAS 5-1 and DAS AB016 in one normal colon tissue and four CRC tissues. These 2 antibodies showed a similar staining pattern, although DAS AB016 showed higher sensitivity. Supplementary Figure 2: negative control using DiAcSpm to mask the reactivity of DAS AB016 toward DiAcSpm. DiAcSpm staining in 8 CRC specimens using DAS AB016 and negative control antibody using the same concentration of DAS AB016 combined with excess DiAcSpm. Supplementary Figure 3: intracellular level of DiAcSpm after treatment. SW480 and Caco-2 cell lines were treated with 0.5 μm DiAcSpm for 24 hours. 10 cm dishes of treated and untreated (control) cells were collected using 100 μL lysis buffer. Concentrations of DiAcSpm in different cell lysates were determined using ELISA. Supplementary Table 1: analysis of DiAcSpm antibody specific using ELISA (first). Supplementary Table 2: analysis of DiAcSpm antibody specific using ELISA (second). Supplementary Table 3: analysis of DiAcSpm antibody specific using ELISA (third). Supplementary Table 4: top upregulated genes and miRNAs after DiAcSpm treatment. Supplementary Table 5: top downregulated genes and miRNAs after DiAcSpm treatment. [file 6665704.f1.doc]

Supplementary Figure 1


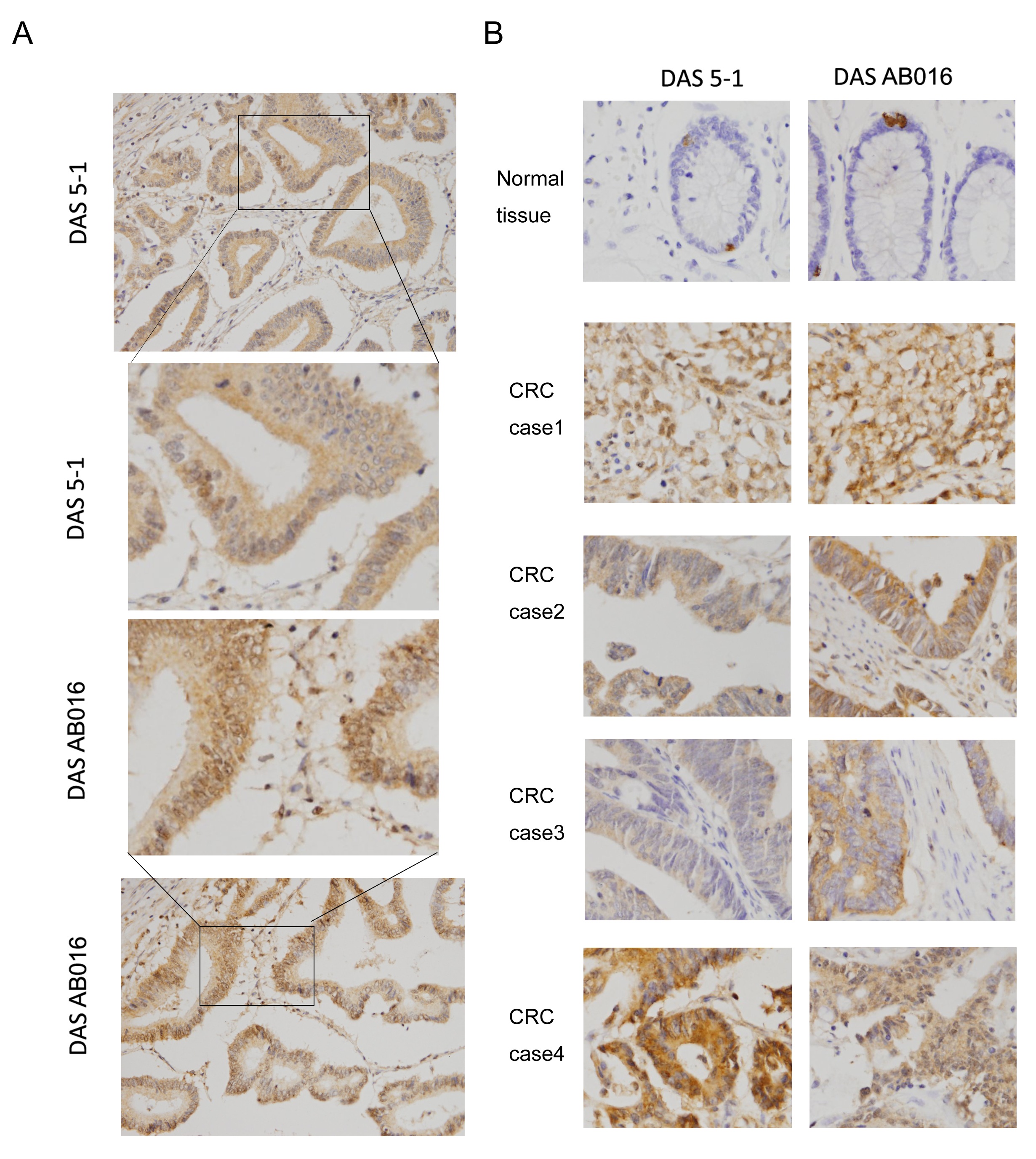


Supplementary Figure 2


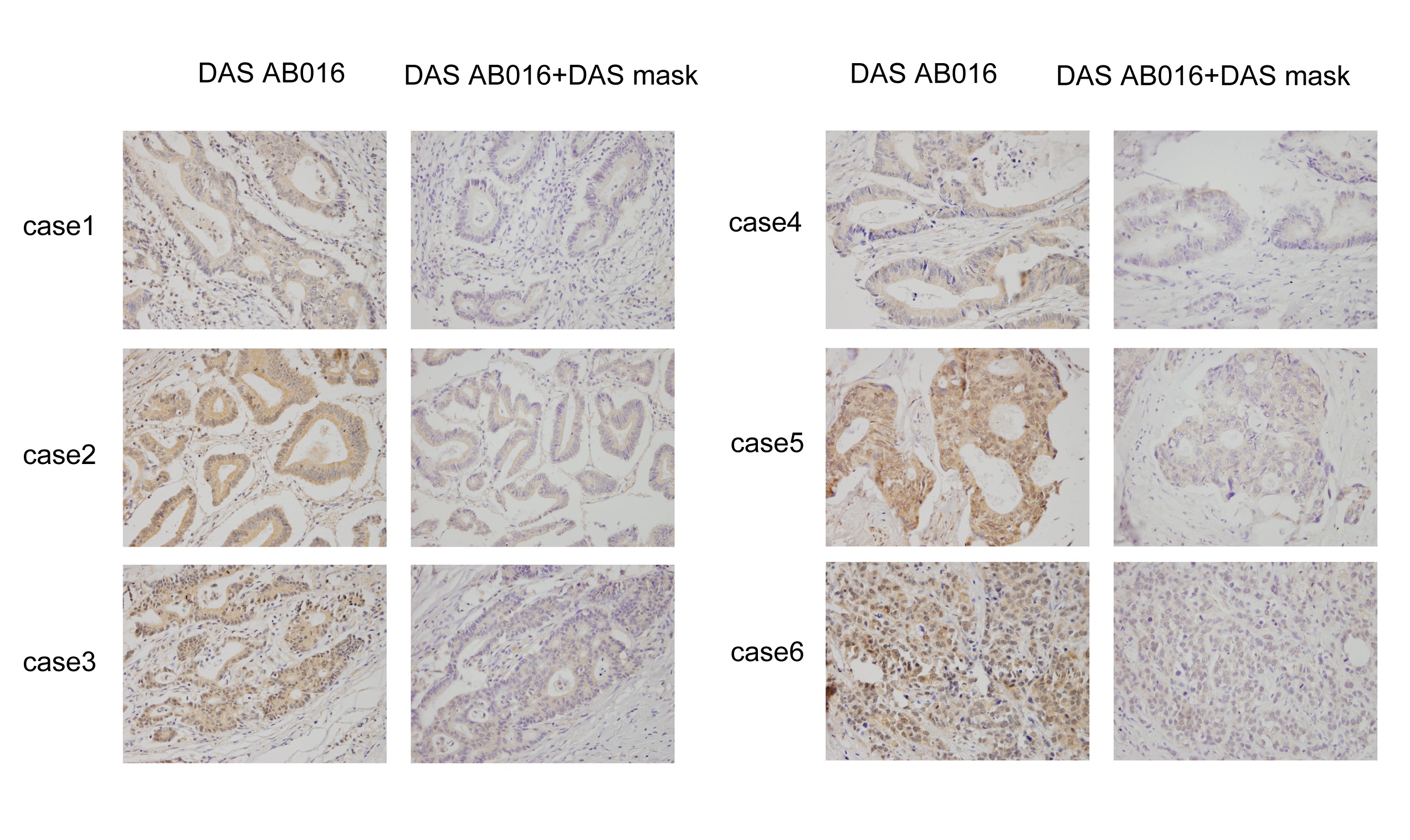


Supplementary Figure 3


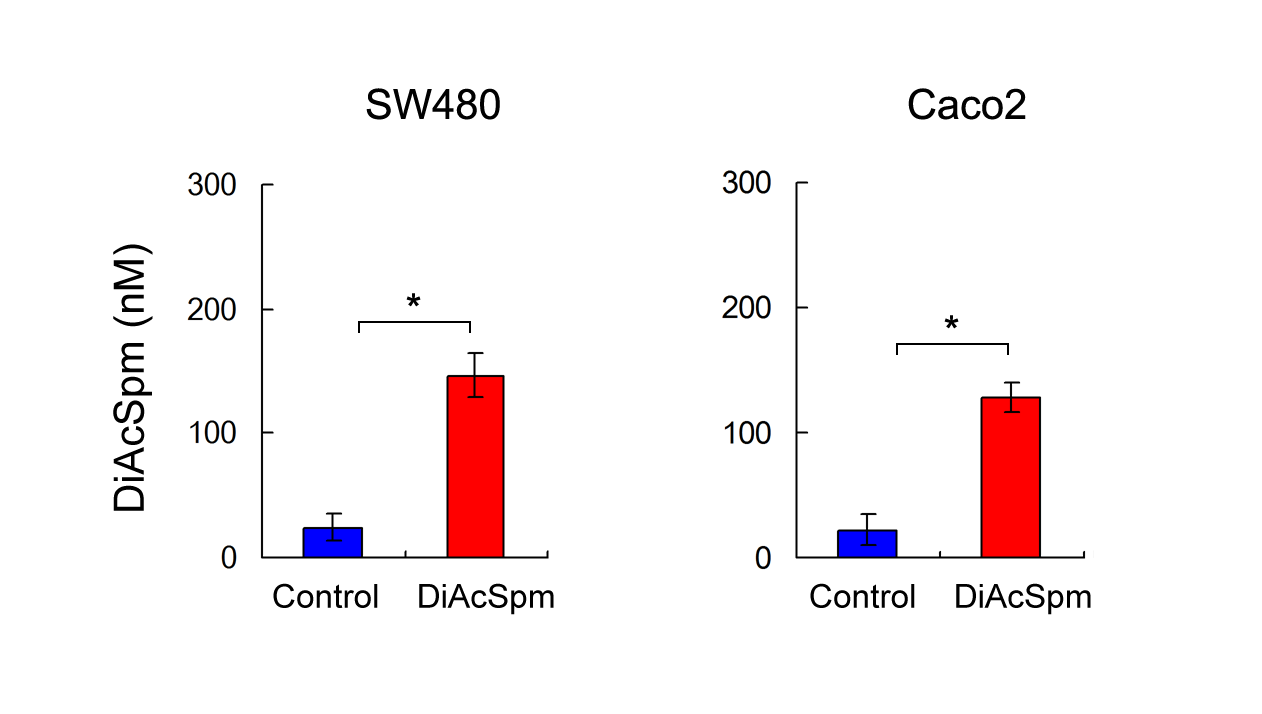


| Supplementary Table1. Analysis of DiAcSpm antibody specific using ELISA (first) | | | | |
| --- | --- | --- | --- | --- |
|  | Repeats | | |  |
| References | 1 | 2 | 3 | Mean (nM) |
| spermine(10μM) | 26.47 nM | 36.73 nM | 37.55 nM | 33.58 |
| putrescine(10μM) | 56.14 nM | 52.76 nM | 57.31 nM | 55.40 |
| diacetylspermidine(5μM) | 77.22 nM | 77.48 nM | 71.82 nM | 75.51 |
| L-tyrosine(2 mM) | 35.38 nM | 31.42 nM | 27.12 nM | 31.31 |

| Supplementary Table2. Analysis of DiAcSpm antibody specific using ELISA (second) | | | | |
| --- | --- | --- | --- | --- |
|  | Repeats | | |  |
| References | 1 | 2 | 3 | Mean (nM) |
| spermine(10μM) | 38.98 nM | 34.21 nM | 28.91 nM | 34.03 |
| putrescine(10μM) | 66.57 nM | 65.94 nM | 58.64 nM | 63.72 |
| diacetylspermidine(5μM) | 69.41 nM | 75.25 nM | 75.85 nM | 73.50 |
| L-tyrosine(2 mM) | 30.53 nM | 27.86 nM | 29.77 nM | 29.39 |

| Supplementary Table3. Analysis of DiAcSpm antibody specific using ELISA (third) | | | | |
| --- | --- | --- | --- | --- |
|  | Repeats | | |  |
| References | 1 | 2 | 3 | Mean (nM) |
| spermine(10μM) | 25.65 nM | 21.71 nM | 38.64 nM | 28.67 |
| putrescine(10μM) | 58.75 nM | 57.66 nM | 67.78 nM | 61.40 |
| diacetylspermidine(5μM) | 65.95 nM | 72.94 nM | 76.25 nM | 71.71 |
| L-tyrosine(2 mM) | 36.52 nM | 35.98 nM | 26.97 nM | 33.16 |

| Supplementary Table 4. Top upregulated genes and miRNAs after DiAcSpm treatment | | | |
| --- | --- | --- | --- |
| gene_name | Control | DiAcSpm | DiAcSpm/Control |
| SNORD117 | 1.155313603 | 4.667137689 | 4.039714999 |
| MIR8075 | 1.097547923 | 4.433780804 | 4.039714999 |
| RF00019 | 1.942562695 | 7.673012995 | 3.949943554 |
| SNORD14E | 1.54947942 | 5.100296873 | 3.291619629 |
| MT-TF | 1.236673716 | 3.8856295 | 3.142000555 |
| MIR106B | 4.283113844 | 13.45754607 | 3.142000555 |
| RN7SL5P | 2.18825754 | 6.752729505 | 3.085893402 |
| RF00019 | 2.262985407 | 6.907149936 | 3.05222911 |
| MIR1244-3 | 4.13194512 | 12.05524715 | 2.917571943 |
| FAM45BP | 1.594958804 | 4.465329327 | 2.799651826 |
| RNU6-1053P | 1.304017334 | 3.511905588 | 2.693143332 |
| RF00019 | 1.357791244 | 3.656726437 | 2.693143332 |
| RNU6-407P | 1.266401449 | 3.410600619 | 2.693143332 |
| RPL39P36 | 1.125690177 | 3.031644995 | 2.693143332 |
| RNY4P10 | 2.743869806 | 6.979099414 | 2.543524258 |
| SPCS2P4 | 5.221813905 | 12.90563706 | 2.471485445 |
| RF00019 | 1.165537617 | 2.790186544 | 2.393905184 |
| RNA5SP311 | 2.270788805 | 5.436053094 | 2.393905184 |
| MT-TI | 3.181298326 | 7.425333392 | 2.334057555 |
| RF01241 | 1.240167144 | 2.894621492 | 2.334057555 |
| CALM2P2 | 1.097547923 | 2.507997223 | 2.285091312 |
| MIR8078 | 1.045283736 | 2.34591577 | 2.24428611 |
| SNORA31 | 1.350828212 | 3.031644995 | 2.24428611 |
| MIR3192 | 1.14030953 | 2.55918084 | 2.24428611 |
| RNU1-106P | 5.353892305 | 11.77535281 | 2.199400388 |
| RNA5SP82 | 4.29475274 | 9.424461613 | 2.194413086 |
| PRKAG2-AS1 | 1.019219051 | 2.236587623 | 2.194413086 |
| RWDD4P1 | 1.165537617 | 2.511167889 | 2.154514666 |
| RF00019 | 2.131161015 | 4.591617662 | 2.154514666 |
| AC093909.6 | 1.246247964 | 2.644376795 | 2.121870504 |
| RNU6-312P | 1.230894866 | 2.578314902 | 2.094667036 |
| AL450124.1 | 1.492416439 | 3.126115518 | 2.094667036 |
| MIR324 | 1.586816274 | 3.323851741 | 2.094667036 |
| RNU6-415P | 1.357791244 | 2.844120562 | 2.094667036 |
| AP000553.4 | 1.855010573 | 3.8856295 | 2.094667036 |
| MIR320E | 2.485014164 | 5.205277255 | 2.094667036 |
| RF00017 | 1.089763895 | 2.236106946 | 2.051918729 |
| AL157871.2 | 1.431584247 | 2.927294895 | 2.044794012 |
| SUMO2P17 | 1.076747126 | 2.196844199 | 2.0402601 |
| AC011462.4 | 1.075548076 | 2.194397825 | 2.0402601 |
| AP000553.7 | 1.488200573 | 3.005953088 | 2.019857499 |
| MIR3907 | 1.162964686 | 2.349022943 | 2.019857499 |
| AP000254.1 | 1.097547923 | 2.197942621 | 2.00259376 |

| Supplementary Table 5. Top downregulated genes and miRNAs after DiAcSpm treatment | | | |
| --- | --- | --- | --- |
| gene_name | Control | DiAcSpm | DiAcSpm/Control |
| RNU7-20P | 5.664763471 | 1.271334998 | 0.224428611 |
| SERF1B | 5.136999126 | 1.162577727 | 0.226314566 |
| HIST2H2AA3 | 4.638089582 | 1.220388969 | 0.263123199 |
| RF00093 | 6.271702415 | 1.689059354 | 0.269314333 |
| RF00019 | 3.377070531 | 1.010548332 | 0.299238148 |
| RNU6-652P | 3.28238631 | 1.104992101 | 0.336642917 |
| MIR26A1 | 5.70154765 | 2.047344672 | 0.359085778 |
| RNU6-722P | 7.176274878 | 2.65268937 | 0.369647124 |
| RNA5SP317 | 2.649253606 | 1.019259955 | 0.384734762 |
| RNU5A-1 | 2.649253606 | 1.019259955 | 0.384734762 |
| RNU6-126P | 2.983625421 | 1.147904415 | 0.384734762 |
| MIR641 | 3.104175943 | 1.194284392 | 0.384734762 |
| SNORD93 | 4.152884031 | 1.597758848 | 0.384734762 |
| RN7SL81P | 3.027718407 | 1.208011799 | 0.398984197 |
| MIR559 | 7.774297785 | 3.284282077 | 0.422453856 |
| MIR579 | 2.687872463 | 1.206470967 | 0.448857222 |
| AC092611.2 | 3.465940808 | 1.555712563 | 0.448857222 |
| RNU6-945P | 3.28238631 | 1.473322801 | 0.448857222 |
| AP001271.1 | 5.114786435 | 2.295808831 | 0.448857222 |
| MIR544B | 2.251380354 | 1.010548332 | 0.448857222 |
| AC116025.2 | 3.468548739 | 1.601365528 | 0.461681714 |
| HMGN1P38 | 3.622270371 | 1.690917505 | 0.466811511 |
| SNORA66 | 6.271702415 | 2.963262025 | 0.472481286 |
| AL161729.4 | 2.398003864 | 1.15915838 | 0.483384701 |
| SRP9P1 | 2.186685516 | 1.057010324 | 0.483384701 |
| AC005696.1 | 3.270345954 | 1.601365528 | 0.489662424 |
| AL591866.1 | 2.189621791 | 1.08111031 | 0.493742944 |
| SNORA12 | 2.687872463 | 1.340523297 | 0.498730247 |
| AC020913.3 | 3.160938017 | 1.576455397 | 0.498730247 |
